# Fluorescent phosphorus dendrimers excited by two photons: synthesis, two-photon absorption properties and biological uses

**DOI:** 10.3762/bjoc.15.221

**Published:** 2019-09-24

**Authors:** Anne-Marie Caminade, Artem Zibarov, Eduardo Cueto Diaz, Aurélien Hameau, Maxime Klausen, Kathleen Moineau-Chane Ching, Jean-Pierre Majoral, Jean-Baptiste Verlhac, Olivier Mongin, Mireille Blanchard-Desce

**Affiliations:** 1 Laboratoire de Chimie de Coordination (LCC), CNRS, 205 Route de Narbonne, BP 44099, 31077 Toulouse Cedex 4, France; 2 LCC-CNRS, Université de Toulouse, CNRS, Toulouse, France; 3 Univ. Bordeaux, ISM (CNRS-UMR5255), Bat A12, 351 Cours de la Libération, 33400 Talence, France; 4 Univ. Rennes, CNRS, ISCR (Institut des Sciences Chimiques de Rennes), UMR 6226, F-35000 Rennes, France

**Keywords:** bioimaging, dendrimer, fluorescence, phosphorus, two-photon absorption

## Abstract

Different types of two-photon absorbing (TPA) fluorophores have been synthesized and specifically functionalized to be incorporated in the structure of phosphorus dendrimers (highly branched macromolecules). The TPA fluorophores were included in the periphery as terminal functions, in the core, or in the branches of the dendrimer structures, respectively. Also the functionalization in two compartments (core and surface, or branches and surface) was achieved. The consequences of the location of the fluorophores on the fluorescence and TPA properties have been studied. Several of these TPA fluorescent dendrimers have water-solubilizing functions as terminal groups, and fluorophores at the core or in the branches. They have been used as fluorescent tools in biology for different purposes, such as tracers for imaging blood vessels of living animals, for determining the phenotype of cells, for deciphering the mechanism of action of anticancer compounds, and for safer photodynamic therapy.

## Introduction

**Figure 1:**
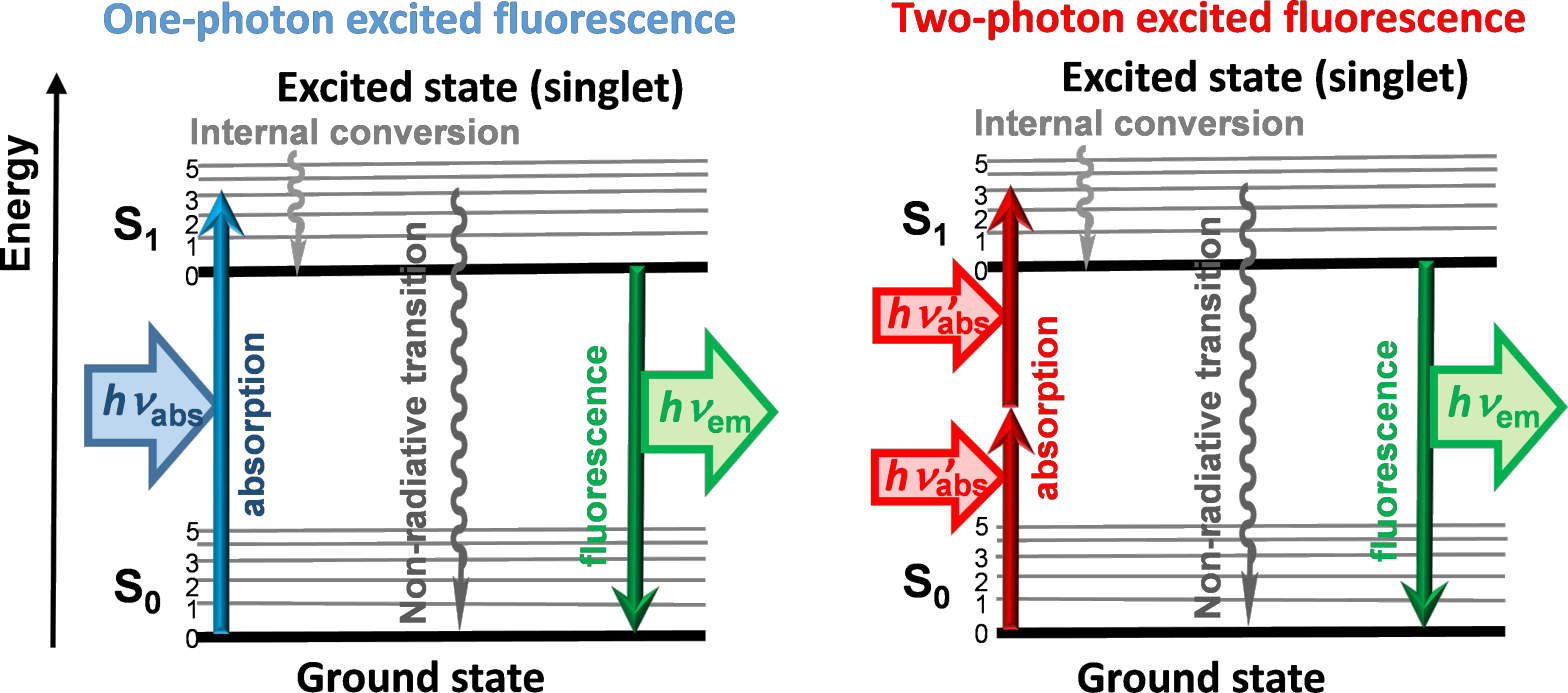
Jablonski-type diagram displaying the classical one-photon excited fluorescence (left), and the less-classical two-photon excited fluorescence (right).

Natural luminescence phenomena such as the bioluminescence of fireflies or of certain marine microorganisms, or the phosphorescence of certain minerals after being exposed to sun light, have fascinated Men for a long time. An important part of luminescence phenomena is due to the influence of light (generally visible or ultra-violet light) on matter, such as for instance a molecule, which can emit light at a wavelength different from that absorbed. The classical Jablonski diagram (Figure 1, left part) represents electronic states of a molecule, and the transitions between these electronic states, depending on the energy. When a molecule absorbs light (a quantum of energy), it goes from a fundamental state (S_0_) to an excited state (S_1_). Depending on the type of molecules, two main ways can be taken to go back to the ground state: either a nonradiative decay of the energy absorbed, or the emission of fluorescence at a longer, less energetic wavelength. A third possibility (not shown in the Figure) concerns an intersystem conversion towards a triplet state (T_1_), from which there will be emission of phosphorescence, having generally a lifetime longer than that of fluorescence. This classical one-photon excited fluorescence has led to the design of numerous types of chemical entities since the XIX century. For instance, fluorescein, which is a widely used fluorescent tracer for many applications, was synthesized for the first time in 1871 [1].

Besides this classical type of one-photon-induced fluorescence, a theoretical work by M. Göppert-Mayer in 1931 [2] predicted the possibility of the simultaneous absorption of two photons (of same or different energy), but this phenomenon was not observable until the advent of lasers. Two-photon excited (TPE) fluorescence is a third-order nonlinear optical process, which was first experimentally observed only in 1961 [3]. It provides intrinsic spatial selectivity in three dimensions, and can be induced at a frequency of half the actual energy gap, thus at longer wavelengths (typically 700–1300 nm), but the fluorescence generally occurs at the same wavelength than when excited with one photon, as illustrated in Figure 1 (right part). These properties have induced widespread popularity of TPE (also named TPA, for two-photon absorption) in the biology community, with on one side, the advent of two-photon excitation fluorescence microscopy [4], even for research clinical uses [5], and on the other side with the discovery of the TPA properties of quantum dots (inorganic nanocrystals [6]), widely used for imaging live cells, for in vivo imaging and diagnostics [7]. Even if the properties of quantum dots have been compared with those of classical organic fluorophores [8], organic fluorophores having giant TPA properties are far less common [9], although there are noticeable exceptions [10] for specifically engineered fluorophores [11]. The main reason is that a single organic fluorophore having TPA properties cannot be as brilliant as a quantum dot, mostly based on its smaller size (or number of delocalized electrons).

**Figure 2:**
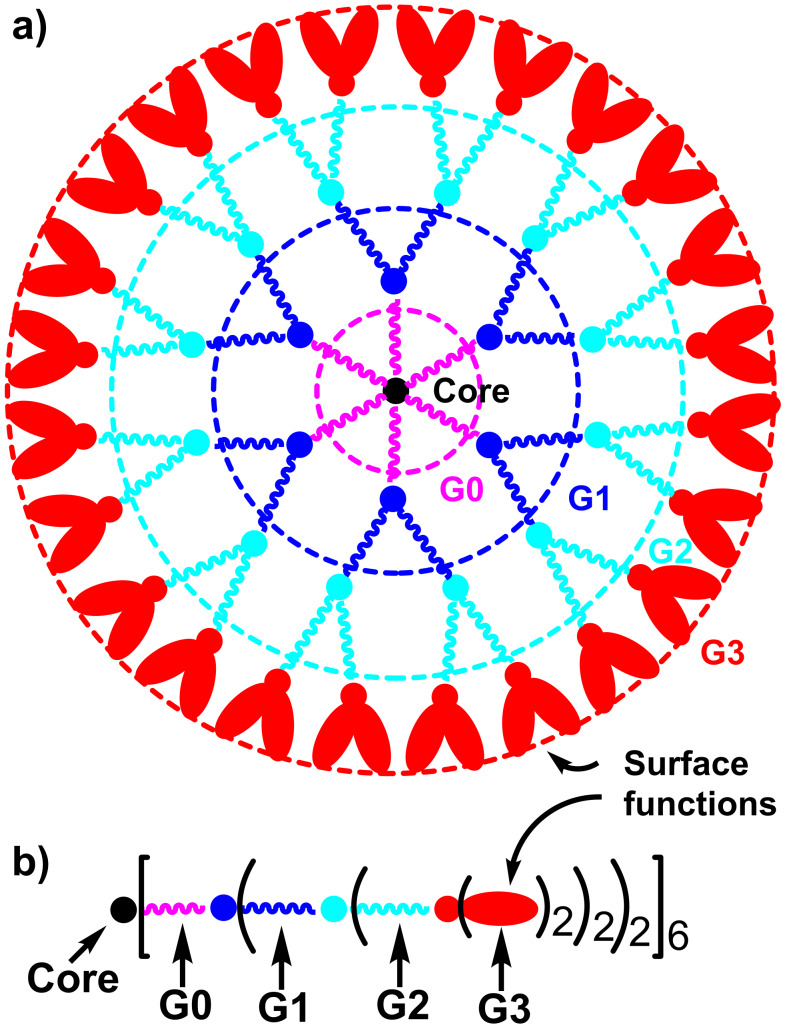
Two ways to represent schematized structures of dendrimers, showing the different generations (layers); a) full structure; b) linear structure with parentheses at each generation; multiplying the numbers associated with the parentheses gives the number of terminal functions (48 in the case of the third generation).

In view of this problem of brilliance, came the idea to gather several organic fluorophores (having TPA properties) in a single molecule. Dendrimers are highly branched macromolecules, which possess many properties [12] due, in particular, to a large number of terminal functions, easily modifiable. A schematized structure of a dendrimer is depicted in Figure 2, showing in particular the generations, i.e., the number of layers, both for the full structure, and for a linear representation of the same dendrimer, which will be mainly used in this review.

Many different types of fluorescent dendrimers (with hundreds of publications), having one or several classical fluorophores in their structure, have been already synthesized [13]. Besides, some types of dendrimers have shown non-traditional intrinsic luminescence, i.e., luminescence in the absence of known fluorophores [14]. Dendrimers functionalized with fluorophores having two-photon absorption properties have been synthesized far less frequently than those bearing classical fluorophores, even if the first examples were proposed in 2000 [15] and 2001 [16]. Among them, conjugated fluorescent dendrimers have been shown to lead to large TPA responses, with coherent coupling contributing to cooperative enhancement of TPA responses [17–21]. Phosphorus-containing dendrimers [22], mainly of the phosphorhydrazone type [23], stand out among all the other types of dendrimers by their properties [24], in particular in biology [25–26] and their highly modular structure [27]. Indeed, different types of modifications, on the terminal functions [28–29], at the core [30] or linked to the core [31], and in the branches [32] have been achieved. In this review, we will gather the syntheses, fluorescence properties, and some biological properties of phosphorhydrazone dendrimers bearing in some part of their structure organic fluorophores especially engineered to demonstrate TPA properties and maintain fluorescence upon confinement within the dendrimeric backbone. The presentation will be organized depending on the location of the fluorophores, either as terminal functions, at the core, in the branches, or at two different locations in the structure of the dendrimers, and will report at the end the biological properties.

## Review

**Scheme 1:**
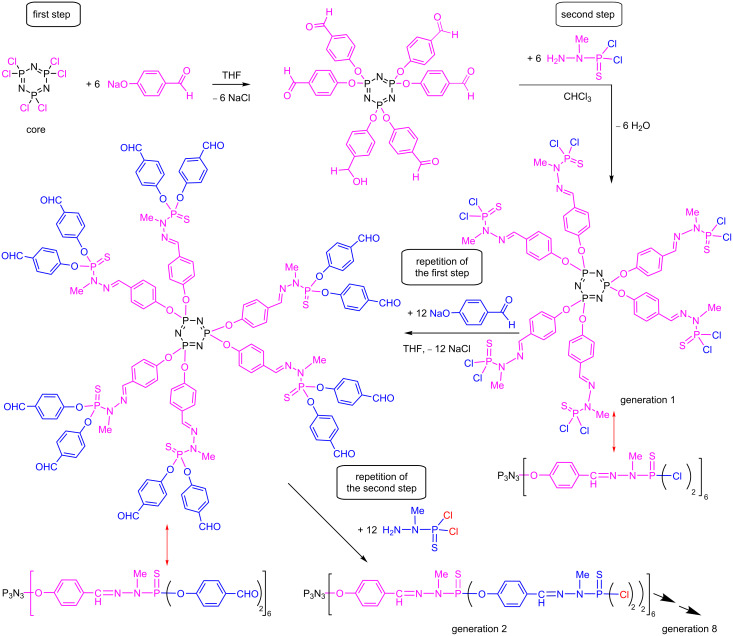
Synthesis of phosphorhydrazone dendrimers, from the core to generation 2. Generation 1 dendrimers with P(S)Cl_2_ or aldehyde terminal functions are represented both as the full structure and in the linear form with parentheses.

The synthesis of phosphorhydrazone dendrimers necessitates two steps to build one generation. Starting from a core having P–Cl functions, such as the hexachlorocyclotriphosphazene N_3_P_3_Cl_6_ [33], the first step is the nucleophilic substitution with 4-hydroxybenzaldehyde in basic conditions. The second step comprises the condensation reaction of the aldehyde terminal functions with the phosphorhydrazide H_2_NNMeP(S)Cl_2_. This reaction affords the first generation (G1) dendrimer, having 12 P–Cl functions, whereas the core had only 6 P–Cl functions. Starting from this first generation, the two-step process can be repeated, as was done from the core, and the second generation is obtained (Scheme 1). The repetition of this two-step process has been carried out up to the eighth generation from the N_3_P_3_Cl_6_ core [34], and up to the twelfth generation from the (S)PCl_3_ core [35]. Thus, to incorporate the TPA fluorophores on the surface, it should have one function being able to react with P–Cl (or aldehyde) functions. For the incorporation of a TPA fluorophore at the core, it should have two identical functions able to react with two N_3_P_3_Cl_6_. To incorporate TPA fluorophores in the branches, these should have two different functions, one able to react with P–Cl functions, the other one being able to react with the NH_2_ group of the phosphorhydrazide, i.e., to replace 4-hydroxybenzaldehyde.

### TPA fluorophores on the surface of dendrimers

**Scheme 2:**
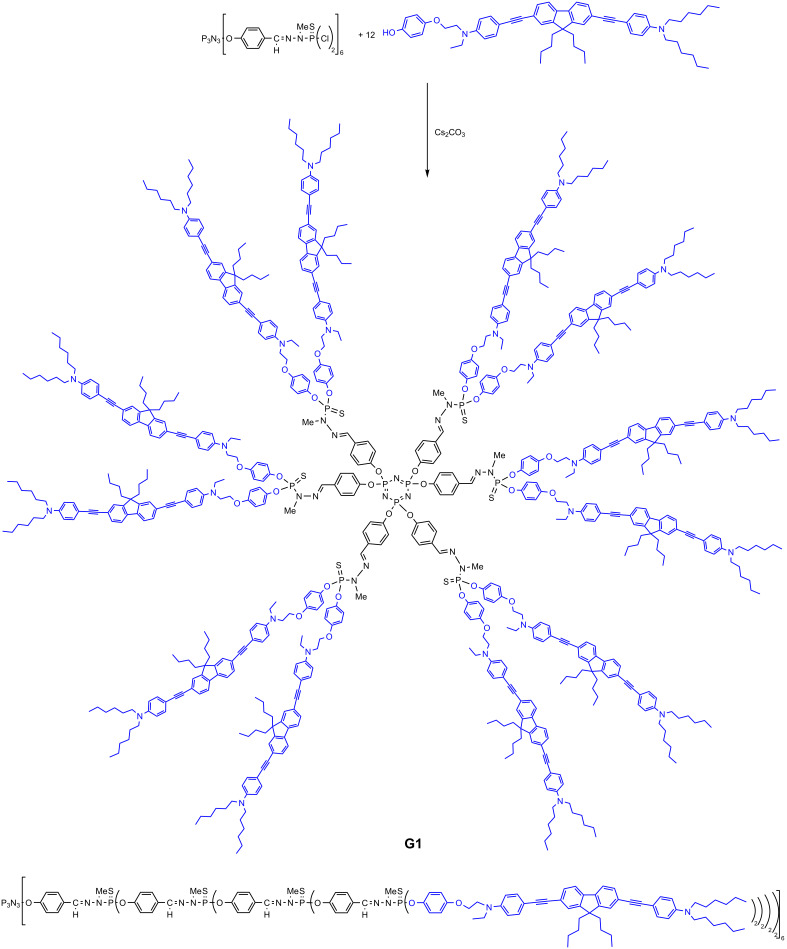
Full structure of the generation 1 dendrimer bearing 12 blue-emitting TPA fluorophores on the surface, and linear structure of the generation 4 dendrimer bearing 96 TPA fluorophores on the surface.

The easiest way to modify the P(S)Cl_2_ terminal functions of the phosphorhydrazone dendrimers consists in using phenols [36], and all the TPA fluorophores on the surface of dendrimers were linked in this way. The very first example of phosphorhydrazone dendrimers functionalized with TPA fluorophores used a blue-emitting quadrupolar fluorophore based on a fluorene core [37–41] and functionalized by a phenol on one side. The synthesis was carried out from generation 1 (12 fluorophores) to generation 4 (96 fluorophores) (Scheme 2). The measurement of the two-photon absorption cross-section, which is a marker of the efficiency of the TPA, displayed a linear increase with the number of fluorophores (Table 1), indicative of an additive behavior. In contrast, the fluorescence properties are only weakly affected. In particular, fluorescence quenching is prevented thanks to the design of the fluorophore [42]. It should be noted that the largest dendrimers, in particular generation 4, display very large two-photon absorption cross-sections (σ_2_ up to 55,900 GM), which are comparable to those measured for the best quantum dots [43]. Thus these “organic nanodots” are fluorescent markers competitive and complementary to quantum dots. This work allowed access to a variety of dendrimers with different emissive colors, by tuning the structure of the quadrupolar fluorophores grafted [44], and not depending on the size, contrarily to quantum dots.

**Table 1:** Photophysical properties of the types of dendrimers shown in Scheme 2 (measured in toluene).

generation	monomer	1	2	3	4

number of fluorophores	1	12	24	48	96
λ_abs,max_/nm	386	385	386	386	386
λ_em,max_/nm	420	423	426	441	445
Φ_f_	0.83	0.75	0.71	0.62	0.48
λ_TPA,max_/nm	702	701	701	701	705
σ_2_^max^ at λ_TPA,max_/GM^a^	765	8,880	17,700	29,800	55,900

^a^GM for Göppert-Mayer, 1 GM = 10^−50^ cm^4^ s photon^−1^.

**Figure 3:**
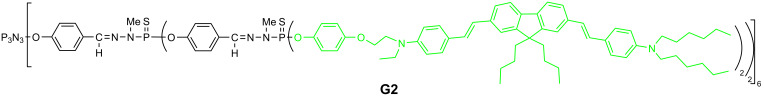
Linear structure of the generation 2 dendrimer bearing 24 green-emitting TPA fluorophores on the surface.

Besides the blue-emitting TPA fluorophore shown in Scheme 2, a related green-emitting fluorophore functionalized by a phenol has been synthesized and grafted to the surface of the second generation dendrimer (Figure 3). A very high TPA cross-section of 35,000 GM was obtained at 740 nm for this dendrimer bearing 24 fluorophores, compared to 1,400 GM for the corresponding monomer and 780 GM for the quantum dot QD518 (CdSe) [45].

In both previous cases, an essential additive effect was observed, depending on the number of fluorophores grafted to the dendrimers. However, an influence of the structure of the dendrimer can be observed in some cases, this effect being either positive or negative. Such effect is named the “dendritic (or dendrimer) effect”, and is frequently observed for catalytic dendrimers, but also for some bioactive dendrimers [46].

**Scheme 3:**
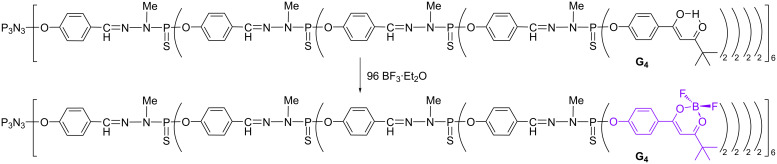
Synthesis of the dioxaborine-functionalized dendrimer of generation 4.

A negative dendrimer effect has been observed for dioxaborine derivatives grafted on the surface of phosphorhydrazone dendrimers. In this case, a β-diketone functionalized by a phenol was first grafted on the surface of the dendrimer [47], then BF_3_ was added to obtain the dioxaborines (Scheme 3). Monomer, dimer, and all generations of the dendrimer from zero (6 terminal groups) to four (96 terminal groups) have been synthesized. Dioxaborine derivatives are known as highly fluorescent tracers [48], and also as two-photon probes [49]. Deceptively, the fluorescence of the dendritic dioxaborines is quenched and dramatically decreased compared to the emission of the isolated monomeric fluorophore, presumably due to interactions between the terminal groups of the dendrimers [50].

**Figure 4:**
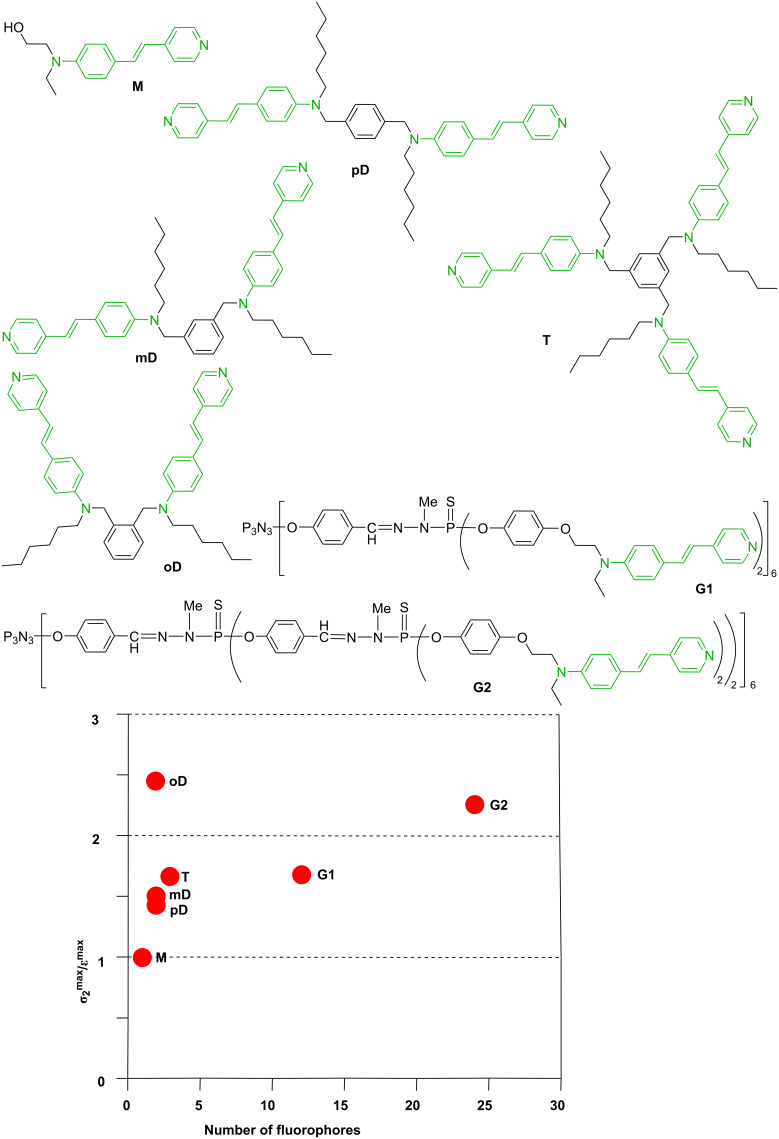
Diverse structures of multistilbazole compounds, and graph of the σ_2_^max^/ε^max^ response, depending on the number of fluorophores.

However, interaction between the terminal groups could be beneficial in some particular cases. Indeed, a cooperative two-photon absorption enhancement has been observed in multichromophoric compounds, thanks to through-space interactions. Modelling suggested that changing the relative orientation/distance of the chromophores would allow cooperative TPA enhancement to be achieved [51]. Thus, multistilbazole molecular structures of different topologies and number of dipolar chromophores, including small dendrimers have been designed to study this possibility. All multichromophoric compounds performed better than the model chromophore. An amplification factor of up to 2.5 per chromophoric subunit was obtained for the σ_2_^max^/ε^max^ response (Figure 4). Interestingly, the response of the dimers increases from the *para-* to the *meta*-position of the stilbazole units, and analogously, the second-generation dendrimer G2 leads to a higher ratio than the first-generation dendrimer, which corresponds in both cases to an increasing proximity of the chromophoric subunits [52].

**Figure 5:**
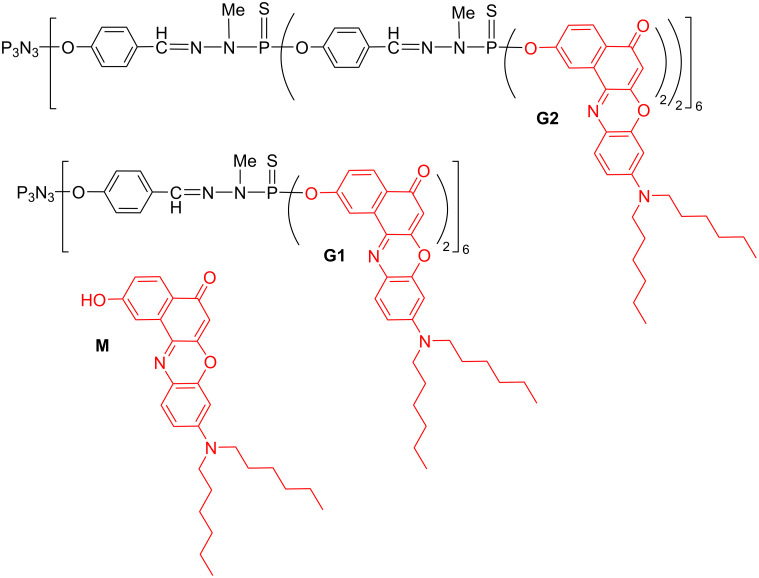
Nile Red derivatives: monomer (M) and two generations of dendrimers.

A second series of compounds concerned derivatives of Nile Red functionalized by a phenol, also grafted to the first and second generation dendrimers (Figure 5). An enhancement of the TPA responses per chromophoric units was observed, but it is less pronounced than for the previous series. Indeed, an increase of TPA of only 20% is obtained for the dimer and 33% for the G1 dendrimer, but a 15% decrease of the TPA response was measured for the G2 dendrimer [53]. In addition, in that case, also the fluorescence quantum yield was found to decrease dramatically with an increasing number of fluorophores.

### TPA fluorophore as core of dendrimers

**Scheme 4:**
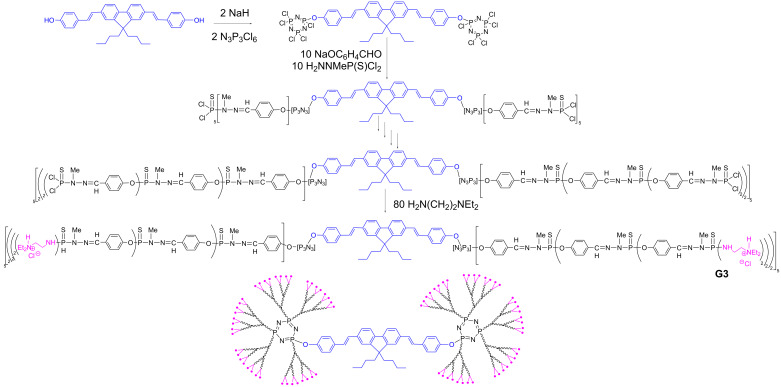
Dumbbell-like dendrimers (third generation) having one TPA fluorophore at the core, and ammonium terminal functions.

None of the previous examples of dendrimers bearing as terminal functions compounds having TPA properties is soluble in water, and thus they cannot be used for biological purposes. Furthermore, many fluorophores are sensitive to the presence of water, which induces in many cases a quenching of the fluorescence [54]. Thus, it could be interesting to use the dendritic structure, which is relatively hydrophobic in the case of phosphorhydrazone dendrimers, to protect the fluorophore, and thus to use the fluorophore as core of the dendrimers. To increase the protective effect, a symmetrical TPA fluorophore functionalized by two phenols was reacted with two equivalents of N_3_P_3_Cl_6_, to afford a core from which 10 branches emanate, instead of 6 as in the previous cases. The growing of the branches was carried out as outlined in Scheme 1, and diethylethylene diamine was grafted in the last step, to ensure the solubility in water [55] of the “dumbbell-like” dendritic structure (Scheme 4). Measurement of the TPA properties showed that the monomeric fluorophore displayed much lower TPA properties in water (σ_2_ = 8 GM, to be compared with 155 GM in ethanol). On the contrary, the fluorophore included as core in all the water-soluble dumbbell-like dendrimers retains a similar TPA response as that of the model monomer in ethanol, as shown by σ_2_ values of 104 GM for G1, 119 for G2, and 127 for G3, in water [56].

**Scheme 5:**
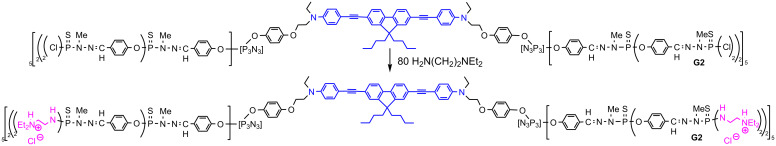
Another example of dumbbell-like dendrimers having one TPA fluorophore at the core, and P(S)Cl_2_ or ammonium terminal functions.

The same type of synthetic method was applied to another TPA fluorophore functionalized by two phenols, but having a longer linker between the fluorophore and the phenol (Scheme 5). Studies of both the dumbbell-like dendrimers of this new series with P(S)Cl_2_ and ammonium terminal functions revealed that the branches are less protective towards the solvents than in the previous case. Indeed, the type of solvent has a large influence on the λ_max_ of the fluorescence for the dendrimer with P(S)Cl_2_ terminal functions, which ranges from 443 nm in AcOEt to 501 nm in DMSO. Furthermore, the quantum yield (Φ_f_) of the dendrimer with ammonium terminal groups was 0.42 in DMSO (to be compared with 0.78 for the corresponding fluorescent monomer of the core), and only 0.075 in water. On the contrary, the TPA response of the fluorophore at the core of the dendrimer is not much affected by the presence of water. Indeed, the TPA response of this dendrimer in water, in the NIR range (700–980 nm) is comparable to that of the corresponding monomeric fluorophore in DMSO [57]. This further confirms that the dendritic architecture provides an “organic-like” environment which preserves the TPA response. Yet, the difference in the structure of the core fluorophore promotes processes which significantly diminish the fluorescence of this dendrimer in water.

### TPA fluorophores in the branches of dendrimers

**Scheme 6:**
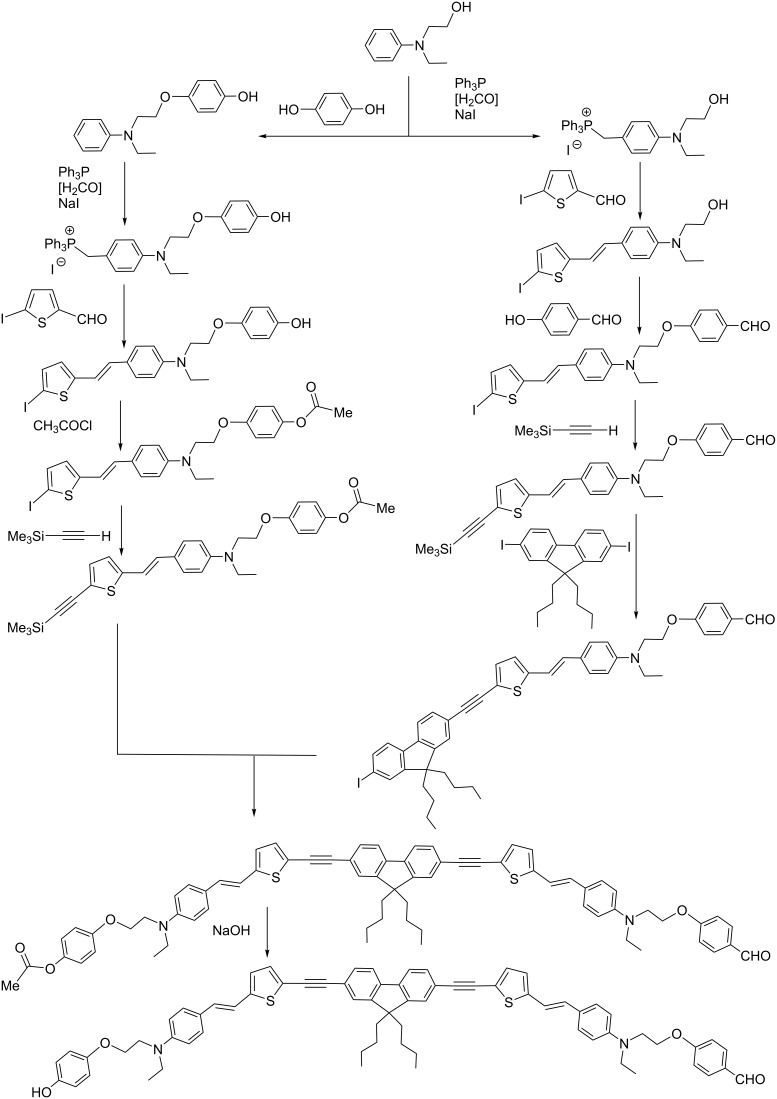
The 12 steps needed to synthesize a sophisticated TPA fluorophore, to be used as branches of dendrimers, in replacement of hydroxybenzaldehyde.

Integrating TPA fluorophores as elements of the internal branches of dendrimers has two advantages, compared to the previous cases: i) the possibility to have water-solubilizing functions as terminal functions, suitable for biological uses (as in the case of TPA fluorophore as core), and ii) a relatively large number of TPA fluorophores included in the structure, which should increase the brilliance, as in the case of TPA fluorophores on the surface. However, there is also an inconvenience, which is the necessity to equip the fluorophore with two different functions, both being compatible with the synthetic process of the dendrimers. In the case of phosphorhydrazone dendrimers, the choice consisted in replacing 4-hydroxybenzaldehyde at one layer of the internal structure by a TPA fluorophore having a phenol on one side and a benzaldehyde on the other side. A detailed synthetic process for one of these bifunctionalized fluorophores is illustrated in Scheme 6. It is a multistep process, as 12 steps are needed to get this highly sophisticated fluorophore [58].

**Scheme 7:**
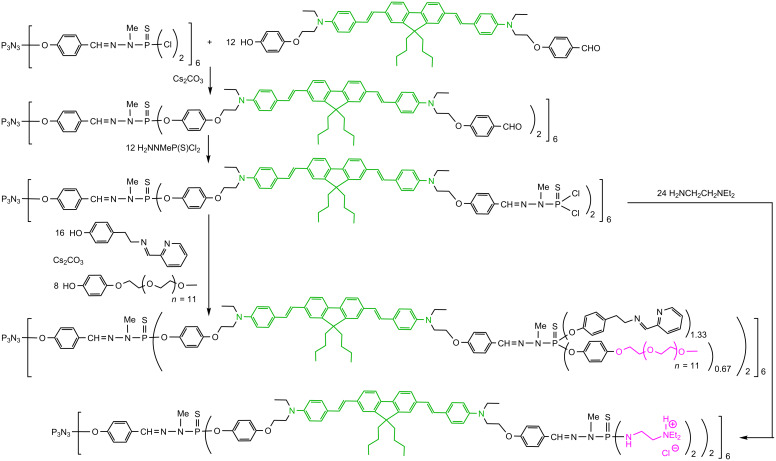
Synthesis of dendrimers having TPA fluorophores as branches and water-solubilizing functions on the surface.

In general, the fluorophores are incorporated at the level of the first generation of the dendrimers. Twelve green-emitting TPA fluorophores were grafted, then reaction with the phosphorhydrazide H_2_NNMeP(S)Cl_2_ afforded the second generation of the dendrimer, with 12 P(S)Cl_2_ terminal functions. These functions were modified in different ways to induce the solubility in water. In one case, phenol PEG (poly(ethylene glycol) was grafted together with pyridine imine phenol, affording dendrimers stochastically functionalized on the surface, but all having precisely 12 fluorophores in the internal structure [59]. Another way to induce solubility in water consists in having positively charged terminal functions [60], in particular ammonium groups. Thus, diethylethylenediamine was directly grafted to the P(S)Cl_2_ terminal functions [61] (Scheme 7). The photo-physical properties, in particular the TPA response of these dendrimers having green TPA fluorophores in the branches have not been determined yet; they have been synthesized for biological purposes that will be explained later.

**Figure 6:**
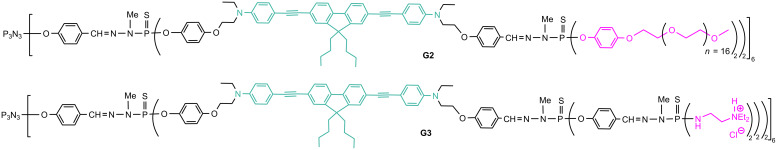
Other types of dendrimers having TPA fluorophores as branches and water-solubilizing functions on the surface.

The same type of synthetic process has been applied to another type of bifunctionalized fluorophore and the dendrimer was also functionalized with water-solubilizing functions. In the first case the synthesis was carried out up to the second generation, then 24 phenol-PEG were grafted. In the second case, the synthesis was carried out up to the third generation, which was functionalized with 48 ammonium groups (Figure 6). Photo-physical studies of both compounds indicated that the fluorophores are more protected by the PEG groups of the second generation G2 than by the ammonium groups of the third generation G3 as indicated by the quantum yields Φ_f_ = 0.24 (G3 ammonium) and 0.39 (G2 PEG). However, the TPA cross-section measurements gave σ_2_^max^ = 13,600 GM for G3 ammonium, and 8,400 GM for G2 PEG. Thus, both compounds exhibit similar performances, the lower quantum yield being counter-balanced by a higher TPA cross-section, and vice versa [45].

### TPA fluorophores at two levels of the dendrimer’s structure

Having in hand the methods to introduce TPA fluorophores either on the surface, at the core, or in the branches of the dendrimers, it seems possible to have them at two different levels, for instance the core and the surface, or at the branches and the surface.

**Figure 7:**
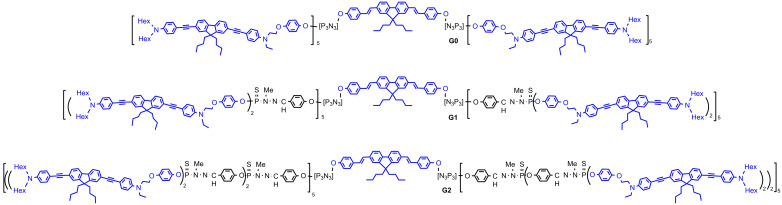
Generations 0, 1, and 2 of dumbbell-like dendrimers having one fluorophore at the core and either 10, 20 or 40 fluorophores on the surface.

In the first example, the core used in Scheme 4 and the terminal functions shown in Scheme 2 were incorporated in dendrimers of different generations (0, 1, and 2), to detect the influence of the distance between the two types of fluorophores and also of a different geometry compared to the symmetrical dendrimers, on their properties (Figure 7). The structure of the dumbbell-like dendrimers has a detrimental influence on the quantum yield, when compared with the symmetrical dendrimers having almost the same number of fluorophores (Table 2). However, the G2 dumbbell-like dendrimer (41 fluorophores) has a higher TPA cross-section than the spherical dendrimer G3 (48 fluorophores). This change might be attributed to changes in the topology, relative orientation, and packing of the fluorophores [62].

**Table 2:** Comparison of the photophysical properties (in toluene) of the spherical dendrimers shown in Scheme 2, with that of the dumbbell-like dendrimers shown in Figure 7.

generation	0	1	1	2	2	3

structure	dumbbell-like	spherical	dumbbell-like	spherical	dumbbell-like	spherical
number of fluorophores	11	12	21	24	41	48
λ_abs,max_/nm	382	385	382	385	384	386
λ_em,max_/nm	443	423	444	426	445	441
Φ_f_	0.44	0.75	0.11	0.71	0.26	0.62
σ_2_^max^ (GM)	7,100	8,800	14,300	17,700	32,800	29,800

**Figure 8:**
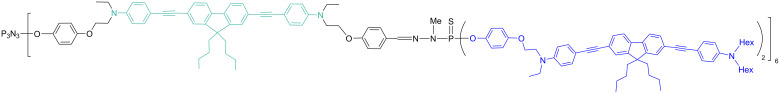
Double layer fluorescent dendrimer.

Another example of fluorophores at two levels concerned the branches and the surface, as depicted in Figure 8. The fluorophore of the branches is the one shown in Figure 6, and the fluorophore of the surface is the one shown in Scheme 2 and Figure 7. This double layer dendrimer, having 18 (6 + 12) fluorophores in its structure, has a TPA cross-section (8,500 GM), which is comparable with that of the first generation dendrimer having only 12 fluorophores on the surface (8,880 GM). It appears that the higher degree of confinement in the double layer dendrimer has a detrimental influence on the TPA properties [63].

### Biological properties of dendrimers having TPA properties

**Figure 9:**
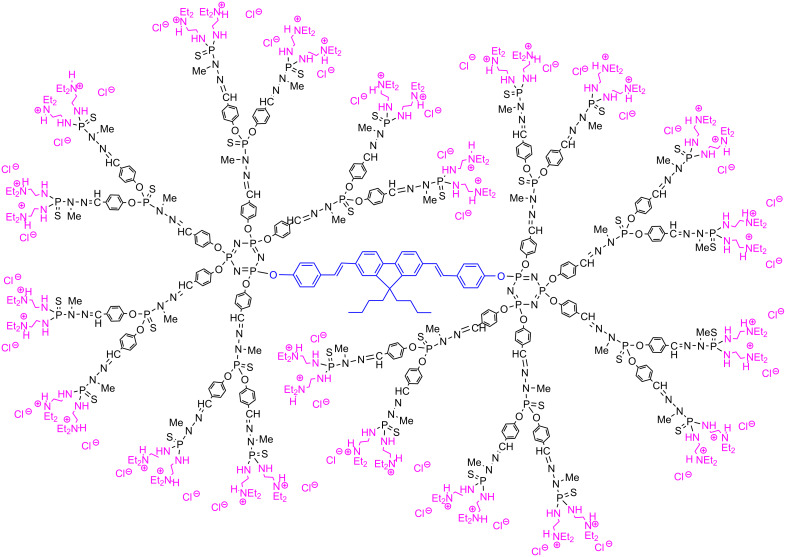
Dumbbell-like dendrimer used for two-photon imaging of the blood vessels of a living rat olfactory bulb.

Most of the water-soluble fluorescent dendrimers shown in the previous paragraphs have been synthesized for diverse biological purposes. The first type of use concerned in vivo imaging with some of the dumbbell-like dendrimers. The second generation of the dendrimer shown in Figure 9 was injected to a rat and the two-photon imaging of the vessels of the living rat olfactory bulb could be obtained [56]. The other dumbbell-like dendrimer shown in Scheme 5 was used for imaging in 3D the blood vessels of the tail of a living Xenopus tadpole [63].

The dendrimer bearing ammonium terminal functions shown in Scheme 7 has been used as fluorescent marker for bone marrow-derived macrophages. It also allowed to determine the phenotype status of these macrophages at different time points after spinal cord injury. This dendrimer has potential uses as a drug/siRNA carrier and phenotype-specific cell tracer, i.e., for enhanced cell therapies combined with monitoring of cell fate and function [61].

**Figure 10:**
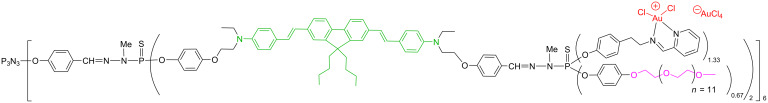
Fluorescent gold complex having high antiproliferative activities against different tumor cell lines.

Dendrimers with pyridine imine terminal functions and their copper complexes have an interesting antiproliferative capacity towards a range of human cancer cell lines, inducing early apoptosis, followed by secondary necrosis [64]. The fluorescent analog having pyridine imine and PEG terminal functions shown in Scheme 7 has been synthesized with the aim of deciphering the mechanism of action of these dendrimers, in particular for monitoring the intracellular penetration. This fluorescent dendrimer avidly binds to the cell membrane during the first 10 min of exposure and after 24 h, it has penetrated the cell, probably by endocytosis, and went in the intracellular space in a high proportion [59]. Besides the copper complexes, the gold complexes of these dendrimers display higher antiproliferative activities, in particular against both KB and HL-60 tumor cell lines (oral epidermoid carcinoma and human leukaemia, respectively), showing IC_50_s (the quantity of a compound necessary to kill 50% of the cells) in the low nanomolar range. The corresponding fluorescent gold complex (Figure 10), keeps in a high level the anti-proliferative activities against KB and HL60: IC_50_s of 60−70 nM and 40−50 nM against KB and HL60, respectively [65]. In these cases, the fluorescence of the dendrimer was not induced by TPA but classically by one photon.

**Figure 11:**
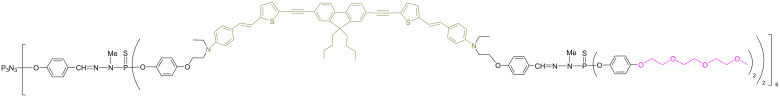
A fluorescent water-soluble dendrimer, applicable for two-photon photodynamic therapy and imaging.

The last example of biological uses concerns a second generation dendrimer built with the fluorophore shown in Scheme 6 at the level of the first generation and having triethylene glycol chains as terminal functions (Figure 11). This dendrimer retains some fluorescence as well as very high TPA cross-sections in a broad range of the NIR (near infra-red) biological spectral window. Furthermore, it displays significant singlet oxygen production, thus this dendrimer combines unique properties for bioimaging and anticancer therapy. Indeed, photodynamic therapy is used in oncology for the treatment of certain types of tumors. It is based on the activation by light of photosensitizers, able to generate singlet oxygen and/or other reactive oxygen species (ROS), to induce the destruction of the targeted tissues [66]. The dendrimer shown in Figure 11 was tested in vitro on human breast cancer cells MCF-7. One-photon absorption induced fluorescence demonstrated that this dendrimer is efficiently internalized after 3 h of incubation, more after 24 h, and was non-toxic at 50 μg mL^−1^ without irradiation. Two-photon irradiation was performed with a confocal microscope; cells were irradiated at 760 nm (in the near IR) by three scans of 1.57 s each at an average power of 80 mW. The percentage of living cells was determined two days after irradiation. 78% cell death was obtained under two-photon irradiation of MCF-7 cells incubated with this dendrimer. It was verified that no cell death was observed in similar irradiation conditions in the absence of the dendrimers, thus revealing the photosensitizing activity of this dendrimer. Importantly, no cell death was observed when the cells were exposed to daylight for 4 h, indicating that this dendrimer is non-toxic under daylight illumination conditions, while promoting cell death upon suitable two-photon irradiation in the NIR [58]. Thus, this dendrimer overcomes one of the common drawbacks of photodynamic therapy medical treatment, which requires patients to avoid daylight after photosensitizer injection.

## Conclusion

**Figure 12:**
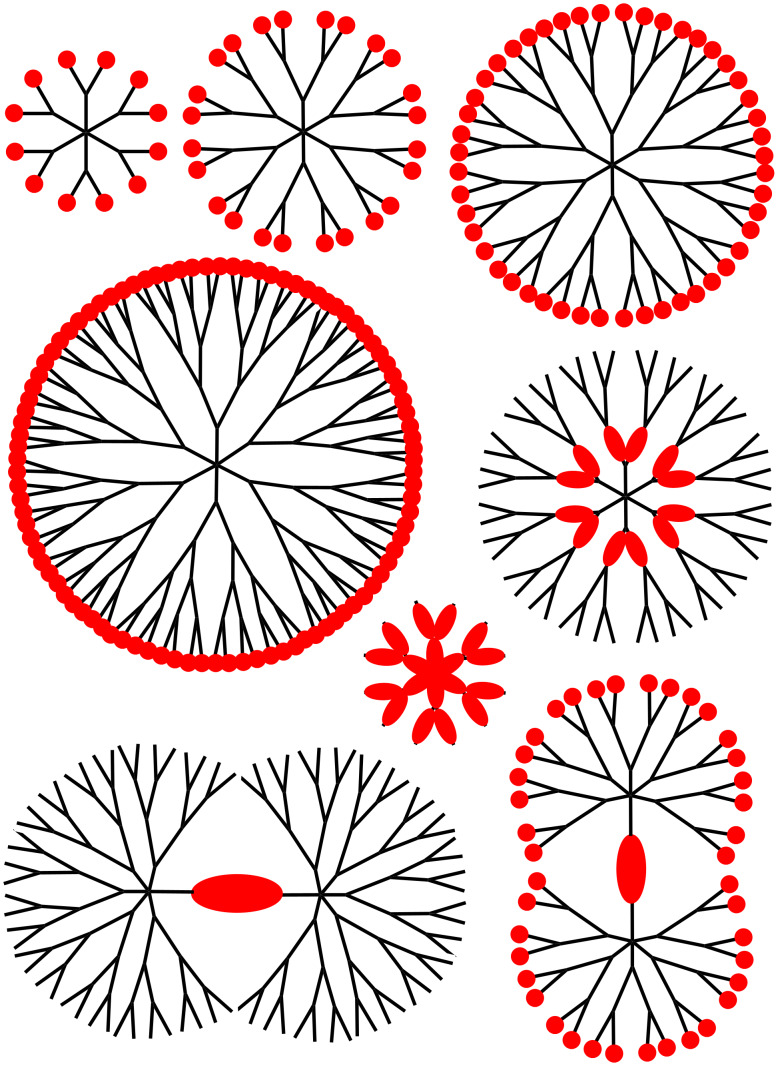
Schematization of the different types of TPA fluorescent phosphorus dendrimers and dendritic structures that have been already synthesized.

The modularity of the synthesis of phosphorhydrazone dendrimers enables the incorporation of TPA fluorophores everywhere in their structure. Classically, the TPA fluorophores can be used as terminal functions of the dendrimers. Less classically, they can be used as core of the dendrimers, leading to dumbbell-like dendrimers, or be incorporated in the branches of the dendrimers. Playing with the specific functionalization of the TPA fluorophores enabled the synthesis of dendrimers having TPA fluorophores at two levels, such as core and surface, or branches and surface (Figure 12). Studies of the TPA properties demonstrated that some of these dendrimers outperform the TPA cross-section response of quantum dots, while having a much lower intrinsic toxicity, as being composed or organic matter instead of heavy metals. The grafting of water-solubilizing functions on the surface of the dendrimers incorporating TPA fluorophores inside their structure has led to interesting properties in biology. Indeed, some of these dendrimers have been used in vivo as tracers for imaging blood vessels of rats and tadpoles, others have been used for determining phenotype of cells, for deciphering mechanism of action of anticancer compounds, and recently for safer photodynamic therapy.

In view of the results already obtained, there is no doubt that other biological properties of these fully organic “nanodots” can be foreseen, thanks to their low toxicity, large color modularity, and high TPA response.
